# Supplementary material for: A comparative study of hematological parameters between hypertensive and normotensive individuals in Harar, eastern Ethiopia
Source: PLoS One. 2021 Dec 7;16(12):e0260751. doi: 10.1371/journal.pone.0260751 (PMC8651120; doi:10.1371/journal.pone.0260751)
Supplement: S1 Annex — (DOCX) [file pone.0260751.s001.docx]

**Annex: Questionnaire** **for hypertension patients and controls**

## A. English Version

Data collection questionnaire designed for “A comparative cross-sectional study of some Hematological parameters of hypertensive and normotensive individuals at public hospitals of Harar, eastern Ethiopia.”

**Study group _______________**

**Code: _________**

| **Sr. No.** | **Questions** | **Possible Responses** |
| --- | --- | --- |
|  | Age (in years) | __________ |
|  | Sex | 1. Male 2. Female |
|  | Educational level | 1. Illiterate 2. Primary (1-8) 3. Secondary (9-10) 4. Preparatory (11-12) 5. Graduate and above |
|  | Occupational status | - 1. Governmental   2. Private   3. Farmer   4. Merchant   5. Student   6. Driver   7. House wife   8. other |
|  | Marital status | - 1. Married   2. Single   3. Divorced   4. Widowed |
|  | Residency | 1. Urban 2. Rural |
|  | Weight: | _____kg |
|  | Height: | ______cm |
|  | BMI (By calculation as: Weight in Kg/Height (cm)^2^) | ______ |
|  | Duration of hypertension since diagnosis in months. (This is for case group only) | _______ |
|  | Blood pressure | Systolic ______mmHg  Diastolic______ mmHg |
|  | Use of drug for blood pressure control. | 1. Yes 2. No |

**Eligibility screening questions (From interview and medical record review)**

| Sr.no | Questions | Possible responses |
| --- | --- | --- |
| 1 | Type of hypertension (for case group only) | 1. Primary hypertension  Secondary hypertension |
| 2 | Pregnancy (for case group only) | 1. Yes 2. No |
| 3 | Medical history of any of disease conditions like diabetes mellitus, cardiac disease, kidney and liver diseases, anaemia, etc.. | Yes 2. No |
| 4 | History of infectious diseases | Yes 2. No |
| 5 | Taking of antibiotics, iron, vitamin B12 or folate supplementations or any other medications | Yes 2. No |
| 6 | Do you drink alcohol | Yes 2. No |
| 7 | If yes, how many days per week | ---------------------- |
| 8 | Are you currently smoking cigarette | Yes 2. No |

## B. Amharic version

| ተ.ቁ | ጥያቄ | መልስ |
| --- | --- | --- |
| 1 | ዕድሜ |  |
| 2 | ጾታ | 1.ወንድ 2. ሴት |
| 4 | የትምህርት ሁኔታ | 1.ያልተማረ 2. የመጀመሪያ ደረጃ (1-8)  3. 2ኛ ደረጃ (9-10) 4. መሰናዶ  5. 12 ክፍል በላይ |
| 5 | የስራ ሁኔታ | 1. የመንግስት ሰራተኛ 2. የግል ሰራተኛ   3.ገበሬ 4. ነጋዴ 5 .ተማሪ 6. ሾፌር |
| 6 | የጋብቻ ሁኔታ | 1. ያላገባ(ባች) 2. ያገባ(ባች) 3. የፈታ(ታች) 4. የሞተችበት(ባት) |
| 7 | መኖሪያ | 1.ከተማ 2. ገጠር |
| 8 | ክብደት | …………… ኪሎ ግራም |
| 9 | ቁመት | …………….. ሲቲ መትር |
| 10 | ቦዲ ማስ ኢዴክስ | ………………. |
| 11 | ግፊቱ ከተገኘ ምን ያህል ጊዜ ሆነው  ( በአመት) | ………………. |
| 12 | የደም ግፊት | ሲስቶሊክ ……………. mmHg  ዳይቶሊክ ……………… mmHg |

**የጥናቱ** **ተሳታፊዎችን መመልመያ መጠይቅ**

| ተ.ቁ | ጥያቄ | መልስ |
| --- | --- | --- |
| **1** | የደም ግፊት አይነት | 1.”ፕራይመሪ” 2. “ስከደሪ” |
| **2** | እርገዘነ | 1.አዎ 2. የለም |
| **3** | ሲጋራ ያጨሳሉን ? | 1.አዎ 2. የለም |
|  | መልሱ አዎ ከሆነ | 1 ከዚህ በፊት 2. አሁንም ድረስ |
| **4** | የአልኮን መጠጥ ይጠጣሉ | 1.አዎ 2. የለም |
|  | መልሶዎ አዎ ከሆነ በሳምንት ስንት ጊዜ | …………………………… |
| **5** | በተላላፊ በሽታዎች ወይም ምልክት | 1. አዎ 2. የለም |
| **6** | ክሮኒክ በሽታዎች እንደ ስኳር የልብ በሽታ የኩላሊት በሽታ የጉበት በሽታ እና የመሳሰሉት አለቦት? | 1.አዎ 2. የለም |
| **7** | የህክምና መዳኒቶች እንደ አቲባዮቲክስ ቢታሚን ቢ 12 ፎሌት ወይም ሌላ መዳሀኒት እየወሰዱ ነው? | 1. አዎ 2. የለም |

## C. 0romiffa questionnaire

Gattiwaan Dhiyaatan

| Lakk | Daaffii | Deebiiwaan deebitaman |
| --- | --- | --- |
| 1 | Umrii(waggaadhan) | _____________ |
| 2 | Saala | 1. Dhiira 2. Dhalaa |
| 4 | Sadarkaa barnootaa | 1. sadarkaalammaffaa(9-10) 2. sadarkaatokkoffaa(1-8) 3. sad. 2ffa (9-10) 4. qophaahina(11-12) 5. kutaa 12 ol |
| 5 | Sadarkaa hojii | 1. Hojjetaa mootummaa 2. hojjetaa dhuunfaa 3. Qonnaan bulaa 4. daldalaa 5. Barataa 6. konkolaachisaa |
| 6 | Iddoo jireenyaa | 1. magaalaa 2. baadiyyaa |
| 7 | haala jireenyaa | 1. kanfuudhe(heerumte) 2. kanhinfuune(hinheerumne) 3. kan hike(hiikte) 4. kanjalaaduhe(duute) |
| 8 | Wfaatina gamaa | _______kg |
| 9 | Dheerina gaamaa | ________cm |
| 10 | Bodii maas indeksi(BMI) | ------------- |
| 11 | Dhiigaa dafqa hanga isin qabee hammam gaye? | ………….. |
| 12 | Gosa dhigaa hanga isaa | _______mmHg |

**Gucha hirmaatoota ittin calalan.**

| Lakk | Daaffii | Deebiiwaan deebitaman |
| --- | --- | --- |
| **1** | Dhiiga danfaa erga beekame yero hangam tae | 1. primary 2. secondary |
| **2** | Uulfa qabdu | 1. eeyyee 2. lakkii |
| **3** | Tamboo nixuuxxaa? | 1. Eeyyee 2. Lakkii |
|  | Yoo eyyee tae | 1. Kanaan dura 2. Amman illee |
| **4** | Dhugaati alkolii nidhuddaa? | 1. Eeyyee 2. Lakkii |
|  | Yoo eyyee tae torbaniti hangam ? | ___________ |
| **5** | Dukkuba daddarboon qabamtee beektu yookiin mallattoolee dhukkuboota daddarboo qabdu. | 1. Eeyyee 2. Lakkii |
| **6** | Dhukuubotta akka onne, sukkarra,dibee tiruu, dhibe kaleefikkf ni qabdaa? | 1. Eeyyee 2. Lakkii |
| **7** | Qorricha fayya kannnen akka antibotics, vitamin B12, fooleeti faa fudhattee beektan ? | 1. Eeyyee 2. Lakkii |
