# Supplementary material for: A comparative study of hematological parameters between hypertensive and normotensive individuals in Harar, eastern Ethiopia
Source: PLoS One. 2021 Dec 7;16(12):e0260751. doi: 10.1371/journal.pone.0260751 (PMC8651120; doi:10.1371/journal.pone.0260751)
Supplement: Attachment — Submitted filename: Response to the reviewers.doc [file pone.0260751.s003.doc]

**Cover letter**

Moges Wordofa

Addis Ababa University

Addis Ababa, Ethiopia

Email: [heranmakmow@gamil.com](mailto:heranmakmow@gamil.com) or moges.wordofa@aau.edu.et

**Date:** May 21, 2021

**To: PLOS ONE**

**Dear Editorial:**

We are glad to write this response to our paper entitled as **“****A comparative study of hematological parameters between hypertensive and normotensive individuals in Harar, eastern Ethiopia”** (Manuscript ID: PONE-D-21-02215**)** which has been requested to review for publication in PLOS ONE journal. We are pleased to have an opportunity to make our paper revised and we have greatly appreciated the editor’s comments and suggestions which were considered as very helpful. In revising the paper, we have carefully considered your comments and suggestions on our revised submission.

**Response to editorial correction**

We have gone through the manuscript to edit and amend some editorial correction. By doing so, we have corrected language error and other topographical errors in the document. We have also prepared the manuscript according to PLOS ONE style requirements. Besides, if there is any error that we missed, we are very ready to correct it again.

**Response to the reviewer’s comment**

**Dear Reviewers:**

We are glad to write this response to our paper entitled as **“A comparative study of hematological parameters between hypertensive and normotensive individuals in Harar, eastern Ethiopia”** (Manuscript ID: PONE-D-21-02215**)** which has been requested to review for publication in PLOS ONE journal. We are pleased to have an opportunity to make our paper revised and we have greatly appreciated your comments and suggestions which were considered as very helpful. In revising the paper, we have carefully considered the comments and suggestions on our revised submission. As instructed, we have attempted to thoroughly explain changes made to all comments and reply to each comment in point-by-point fashion as follows:

**Response to Reviewer#1**

**Comment#1*.*** “Authors did not show how they calculated the sample size. They should explain how they got the sample size.**”**

**Response#1**: The sample size was determined from a study in Indian based on mean and variance of hemoglobin. We used 95% CI and power of 0.8 as well as based on formula for double population of equal size mean. However, we enrolled additional participants other than the one we obtained using the formula. The purpose was to increase presentiveness of the sample to the general population.

**Comment #2. “**Authors did not state the number of males and female in both cases and controls. This should be stated in the manuscript**.**”

**Response #2**: The total number of males and females in both normotensive and hypertensive group were equal and it was 39 and 63, respectively. we have already included this in the result section of the manuscript in a previous submission.

**Comment #3**. “The sampling techniques was not well described in the methodology. The mention of convenient sampling is not enough.”

**Response #3**. As suggested by the reviewer, we have elaborated the technique in the methodology section.

**Comment#4**. “The criteria for selecting the controls are not clearly described in the methodology section. Authors must describe this”

**Response#4.** As suggested by the reviewer**,** we have clarified the donor selection criteria in methodology part of the manuscript**.**

**Comment**#5. “The operational definitions for the cases (hypertensives) and controls (normotensive) are not stated clearly stated. Authors must describe this**”**

**Response#5.** As suggested by the reviewer, we have included the definition of normotensive and hypertensive under operational definition in methodology section

**Response to Reviewer#2**

**Comment#1**: “While appreciating the challenge of preparing a manuscript in the English language for many authors, the paper should be carefully edited by a native English-speaking editor”

**Response#1:** The manuscript is thoroughly revised by native English writer in the field and linguistic errors are corrected

**Comments#2**: “No information about the physical activity records”.

**Response#2:** we thought it is irrelevant to the study

**Comment#3**: “Please explain more about the implication of this study results. How can the study results help clinically?”

**Response#3:** As suggested by the reviewer, the significance of the study is addressed in the introduction part of the manuscript.

**Comment#4:** “The Discussion would be improved by detailing more specific implications for future studies”

**Response#4:** It has been included as it could be used as a baseline for performing further advanced studies including lipid profile and others

**Comment#5:** “The limitation of the study should be fully explained in the discussion section”

**Response#5:** We have included the limitation of the study in the discussion

**Comment#6: “**Please describe more about the novelty of manuscript in the introduction. It is very important for readers to know about this novelty.”

**Response#6:** We have tried to include novelty and necessity of this study among hypertensive patients in the country and worldwide.

Looking forward to hearing from you. Thank you again for your consideration!

Sincerely,

Moges Wordofa
