# Supplementary material for: A comparative study of hematological parameters between hypertensive and normotensive individuals in Harar, eastern Ethiopia
Source: PLoS One. 2021 Dec 7;16(12):e0260751. doi: 10.1371/journal.pone.0260751 (PMC8651120; doi:10.1371/journal.pone.0260751)
Supplement: Attachment — Submitted filename: Response to the reviewers.doc [file pone.0260751.s004.doc]

**Cover letter**

Moges Wordofa

Addis Ababa University

Addis Ababa, Ethiopia

Email: [heranmakmow@gamil.com](mailto:heranmakmow@gamil.com) or moges.wordofa@aau.edu.et

**Date:** Oct 15, 2021

**To: PLOS ONE**

**Response to the reviewer’s comment**

**Dear Reviewers:**

We are glad to write this response to our paper entitled as **“A comparative study of hematological parameters between hypertensive and normotensive individuals in Harar, eastern Ethiopia”** (Manuscript ID: PONE-D-21-02215**)** which has been requested for further revision for publication in PLOS ONE journal. As suggested, we have attempted to separately analyze hematological parameters of males and females among control and hypertensive patient.

**Response to Reviewer#1**

**Comment*.*** “My comment is that when dealing with hematological parameters, authors will have to separate males from females in the analysis. hematological parameters for males and female cannot be analyzed together they must be separated.**”**

**Response**: In addition to comparing the overall hematological parameters between hypertensive and control group, as shown in the result section of the manuscript, we have separately analyzed the parameters by sex between the two groups (normotensive and hypertensive).

Looking forward to hearing from you. Thank you again for your consideration!

Sincerely
